# Applications of Isothermal Titration Calorimetry in Biophysical Studies of G-quadruplexes

**DOI:** 10.3390/ijms10072935

**Published:** 2009-07-02

**Authors:** Bruno Pagano, Carlo Andrea Mattia, Concetta Giancola

**Affiliations:** 1 Dipartimento di Scienze Farmaceutiche, Università di Salerno, Via Ponte Don Melillo, 84084, Fisciano (SA), Italy; E-Mail: mattia@unisa.it (C.M.); 2 Dipartimento di Chimica, Università di Napoli Federico II, via Cintia, 80126, Napoli, Italy

**Keywords:** G-quadruplex, isothermal titration calorimetry, thermodynamic, drug

## Abstract

G-quadruplexes are higher-order nucleic acids structures formed by G-rich sequences that are stabilized by tetrads of hydrogen-bonded guanine bases. Recently, there has been growing interest in the study of G-quadruplexes because of their possible involvement in many biological processes. Isothermal titration calorimetry (ITC) has been proven to be a useful tool to study the energetic aspects of G-quadruplex interactions. Particularly, ITC has been applied many times to determine the thermodynamic properties of drug-quadruplex interactions to screening among various drugs and to address drug design. In the present review, we will focus on the ITC studies of G-quadruplex structures and their interaction with proteins and drugs and the most significant results will be discussed.

## 1. Introduction

Isothermal titration calorimetry (ITC) is a valuable method aimed at characterizing the energetics of molecular interactions [1,2]. ITC gives direct thermodynamic information; in particular, it is the only technique that directly measures the binding enthalpy and it is a high-accuracy method for measuring binding affinities and stoichiometry [3]. Moreover, ITC allows dissecting the free energy of binding into enthalpic and entropic components, revealing the overall nature of the forces that drive the binding reaction. Thanks to its versatility and precision, ITC has been proven to be an useful tool to study the energetic aspects of G-quadruplexes’ interactions with other biomolecules, including small ligands [4].

In recent years, G-quadruplexes have been the topic of thousands of papers because of their possible involvement in many biological processes [5]. Indeed, DNA sequences that can form G-quadruplex structures are widely found throughout the genome and are located in biologically relevant regions [6]. Such DNA sequences are found in the promoter regions of a number of genes and oncogenes and in other parts of the genome, especially in the telomeres. Indeed, in most eukaryotic organisms the telomeric DNA is organized in tandem repeats of short G-rich sequences [d(T_2_AG_3_) in humans] [7–10] that were shown to form G-quadruplex structures able to inhibit *in vitro* the activity of telomerase [11], the enzyme (active in about 85% of cancer cells) that adds the telomeric repeats to the ends of chromosomes favoring the proliferation of cancer cells [12]. On the other hand, it has been hypothesized that the formation of G-quadruplex structures in the promoter region of some oncogenes could play an important role in regulating the transcription of the corresponding gene [13]. G-quadruplex structures from both telomeric and promoter regions have thus emerged as potential targets for anticancer drug development [14]. G-quadruplex molecules are also important because they have been implemented in the design of novel aptamers aimed at binding and inhibiting specific proteins [15].

**Figure 1.**
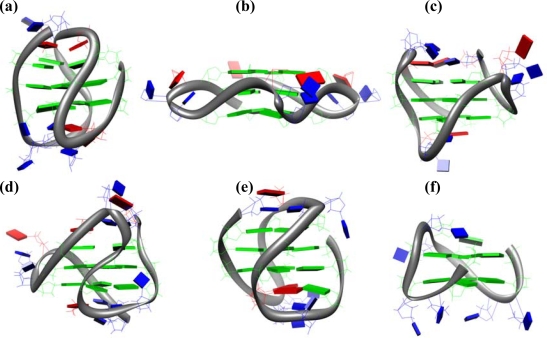
(a) NMR structure of the anti-parallel G-quadruplex formed by the d[AG_3_(T_2_AG_3_)_3_] sequence in Na+ solution (pdb code: 143D). (b) Crystal structure of the parallel G-quadruplex formed by the d[AG_3_(T_2_AG_3_)_3_] sequence in the presence of K+ (pdb code:1KF1). (c) NMR structure of the G-quadruplex formed by the d[TAG_3_(T_2_AG_3_)_3_] sequence (hybrid-1) in K^+^ solution (pdb code: 2JSM). (d) NMR structure of the G-quadruplex formed by the d[TAG_3_(T_2_AG_3_)_3_TT] sequence (hybrid -2) in K+ solution (pdb code: 2JSL). (e) NMR structure of the antiparallel G-quadruplex formed by the d[G_3_(T_2_AG_3_)_3_T] sequence in K+ solution (pdb code: 2KF8). (f) NMR structure of the thrombin binding aptamer (TBA) (pdb code: 1RDE).

G-quadruplexes are four-stranded nucleic acids structures with a core of two or more guanine tetrads stacked upon each other, connected by lateral, diagonal, or external loops [16]. A G-tetrad consists of a planar arrangement of four guanine bases associated through a cyclic array of Hoogsteen-like hydrogen bonds in which each guanine both accepts and donates two hydrogen bonds [17]. G-quadruplexes structures are formed by one, two or four strands of G-rich sequences. A one-stranded structure yields a unimolecular G-quadruplex, two strands produce a bimolecular G-quadruplex, while four separate strands produce a tetramolecular G-quadruplex. Quadruplex structures are designated as parallel when all the strands are parallel each other, and as anti-parallel when at least one of the four strands is anti-parallel to the others. This type of topology is found in the majority of bimolecular and in many unimolecular quadruplex structures determined so far, while the tetramolecular quadruplexes usually are in a parallel orientation. Both length and composition of the loops connecting the G-tetrads are some of the key elements of G-quadruplexes, indeed, they may determine the topology of the molecules and have effect on their thermodynamic stability. Last but not least, the loops may have effect on the molecular recognition of the G-quadruplexes and, thereby, they may determine their functionality [18]. Stacking of G-tetrads produces a cylindrical central cavity, lined with the guanine O6 carbonyl oxygens, that is a specific binding site for metal ions. The coordination of metal ions, preferentially Na^+^ and K^+^, between the adjacent tetrads provides both thermodynamic and kinetic stability to the G-quadruplex structure [19]. All quadruplex structures have four grooves, defined as the cavities bounded by the phosphodiester backbones, whose dimensions are variable, depending on the overall topology of the quadruplex. Particularly, the varying of the grooves widths is a consequence of the glycosidic torsion angles [20]. Quadruplex structures have been shown to possess a surprising structural polymorphism depending on many factors [21]. For example, the single-stranded human telomeric G-rich sequences have been shown to adopt *in vitro* five different G-quadruplex structures depending on the length of the sequence and experimental conditions. The sequence d[AG_3_(T_2_AG_3_)_3_] in Na+ solution forms an anti-parallel basket-type structure involving three G-tetrad layers with a diagonal TTA loop at one end and two lateral TTA loops at the other end (Figure 1a) [22]. The same sequence forms a parallel structure with three symmetrical propeller TTA loops in a K^+^-containing crystal (Figure 1b) [23]. On the other hand, telomeric sequences in K^+^ solution have been shown to form, depending on the presence of flanking bases, two distinct but related hybrid-type structures, known as hybrid-1 and hybrid-2, with the so-called (3+1) conformation (Figure 1c-d) [24–29]. These hybrid structures have three strands oriented in one direction and the fourth in the opposite direction, that are connected by a double-chain-reversal side loop and two lateral loops. Finally, it has recently been reported the structure of a new intramolecular G-quadruplex conformation formed by the human telomeric d[G_3_(T_2_AG_3_)_3_T] sequence in K+ solution (Figure 1e) [30]. This is an antiparallel basket-type G-quadruplex involving only two layers of G-tetrads.

The knowledge of G-quadruplex structures (topology of the molecule, location and length of the loops, groove widths) is essential for a rational design of small molecules that could selectively bind to these structures to specifically modulate gene transcription or to inhibit telomerase, as well as for the developing of aptamers that can selectively bind to particular proteins. Recent reviews present detailed information about the ITC theory, instrumentation and what concerns about practical aspects of performing ITC experiments [31–33]. In the present review, we will focus on the ITC studies of G-quadruplex structures and their interaction with proteins and drugs with only a limited discussion of the theory.

Principally four types of interactions have been investigated for quadruplex-forming oligonucleotides by ITC, these comprise the interaction with: (a) small molecules; (b) proteins; (c) ions; (d) complementary strands. Above all, ITC has been applied many times to understand the thermodynamic proprieties of drug-quadruplex interactions to screening among various drugs and to address drug design.

In ITC, one component of the complex (for example, a quadruplex molecule) is present in the sample cell of calorimeter, and the second component (for example, a drug) is slowly added in an incremental stepwise fashion. Since all binding events give rise to evolution or absorption of heat (a change in enthalpy), the analysis of these extremely small thermal effects arising from the binding allows a full thermodynamic characterization of the reaction and provides fundamental information about the molecular interactions driving the process. In a typical ligand-quadruplex titration, the chemical reaction generated by each injection either releases or absorbs a certain amount of heat (q_i_) proportional to the amount of ligand that binds to the molecule in a particular injection (V×ΔL_i_) and the characteristic binding enthalpy (ΔH°) for the reaction:

$${\text{q}}_{\text{i}}=\text{V}\times \Delta {\text{L}}_{\text{i}}\times \Delta \text{H}{}^{\circ}$$

where V is the volume of the reaction cell and ΔL_i_ is the increase in the concentration of bound ligand after the i^th^ injection [3]. The heat after each injection is therefore obtained by calculating the area under each peak. Because the amount of uncomplexed quadruplex available progressively decreases after each successive injection, the magnitude of the peaks becomes progressively smaller until complete saturation is achieved. Once this situation is reached, subsequent injections produce similar peaks corresponding to the dilution or mechanical effects that need to be subtracted from all the injection peaks before the analysis.

The plot of the heat of each injection as a function of the drug/quadruplex molar ratio usually produces a curve with a sigmoidal shape that facilitates the estimation of the midpoint of the reaction process, and thus the stoichiometry of the binding reaction. The binding constant (K_b_) and ΔH° are calculated by iterative approximation. A value for K_b_ is initially estimated, then the concentration of bound complex is calculated for each injection. In combination with the measured heat, these values are used to determine the average of ΔH°. The ΔH° and the calculated concentration are then used to determine an expected heat per injection, and the error square sum between the measured and expected heat for each peak is calculated. The value of K_b_ is then adjusted and the process repeated until a minimum error square sum is obtained. The values of K_b_, ΔH° and stoichiometry can often be calculated from a single experiment as long as the concentration of both quadruplex and ligand are accurately known and chosen so that:

$$1<{\text{K}}_{\text{b}}[\text{M}]<1000$$

where [M] is the total concentration of molecule in the sample cell titrated by ligand [34]. If the concentrations are not within this range, the curvature of the titration plot can be so weak as to be almost linear, or so strong as to produce a step-like profile. In these cases K_b_ may not be estimated accurately. Typically, quadruplex concentrations in the order of 10–100 μM are used, allowing K_b_ values in the range 10^4^ – 10^8^ M^−1^ to be accurately estimated.

Since temperature (T) is held constant throughout the entire experiment, the free energy (ΔG°) of the binding reaction can be determined by:

$$\Delta \text{G}{}^{\circ}=-\text{RT}\text{ln}{\text{K}}_{\text{b}}$$

where R is the gas constant.

ITC directly measures ΔH°, so the change in entropy (ΔS°) can be determined by:

ΔS°=(ΔH°−ΔG°)/T

Quantification of these thermodynamic parameters reveals the energetics of the physical processes involved in the binding reaction.

## 2. Small Molecules Interactions

The interactions between small molecules and G-quadruplex structures are largely described in ITC literature, thanks to the great current interest in developing ligands that can bind selectively to G-quadruplexes [35,36]. Indeed, many small molecules that bind to quadruplexes have proven to be effective therapeutic agents, although the exact mode of binding and nature of thermodynamic forces that regulate DNA–ligand interactions are often poorly understood. However, it is important in characterizing and optimizing the drug-target interactions to reveal the thermodynamic nature of the forces that drive the drug-quadruplex binding reaction. A variety of small molecules have been devised to bind and stabilize G-quadruplex structures, ranging from porphyrins to distamycins, acridines, and other polycyclic systems [4]. A number of these molecules capable of stabilizing G-quadruplexes are characterized by a core with a large π-surface that favor stacking interactions with the G-tetrads and, in several cases, they contain side chains that could interact with the grooves of the G-quadruplex structures. On the other hand, some molecules are devised to bind to the grooves of the quadruplex, while other molecules were found to interact with the loops.

**Figure 2.**
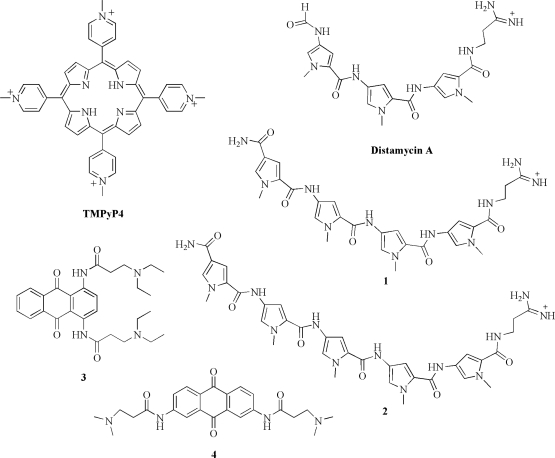
Chemical structures of quadruplex-interactive compounds studied by ITC.

Among all, cationic porphyrins are, from a calorimetric point of view, the most investigated quadruplex-interactive drugs. They are known to bind to and stabilize different types of G-quadruplexes and, in some cases, to facilitate G-quadruplex formation [37]. Particularly, the cationic *meso*-tetrakis-(*N*-methyl-4-pyridyl)-porphyrin (TMPyP4, Figure 2) has been the subject of extensive investigations since it induces telomerase inhibition upon binding to telomeric DNA quadruplexes [38]. Furthermore, TMPyP4 has been shown to downregulate the expression of the *c-myc* oncogene by stabilizing the G-quadruplex structure adopted by the guanine-rich segment of the P1 promoter of the gene [39].

Haq *et al.* were the first to investigate by ITC the thermodynamics for binding of TMPyP4 to three biologically significant DNA G-quadruplex structures: the one formed by the 15-mer d(G_2_T_2_G_2_TGTG_2_T_2_G_2_), known as thrombin binding aptamer (TBA, Figure 1f) (see next section); the one formed by the single-stranded 22-mer human telomeric sequence d[AG_3_(T_2_AG_3_)_3_]; and the tetramolecular [d(T_4_G_4_)]_4_ structure formed by 8-mer strands from the *Oxytricha* telomere [40]. To establish any dependence of binding behavior on the metal ion present in solution, they performed the experiments in two different buffered solutions containing either K^+^ or Na^+^. They determined binding stoichiometries of 1:1, 2:1, and 3:1 for d(G_2_T_2_G_2_TGTG_2_T_2_G_2_), d[AG_3_(T_2_AG_3_)_3_], and [d(T_4_G_4_)]_4_, respectively. All the interactions were found to be exothermic and the binding stoichiometries as obtained resulted to be unaltered for solutions containing either Na^+^ or K^+^ (Table 1). However, nondegenerate sites were found for the 2:1 and 3:1 complexes where the first porphyrin binds with 20–40-fold greater affinity than any subsequent ligand. In fact, the interaction with d[AG_3_(T_2_AG_3_)_3_] in K+ was characterized by a single binding event with an affinity of 2.8 x 10^4^ M^−1^, whereas the binding in Na^+^ solution was characterized by an initial binding event (K_b_ = 3.3 x 10^4^ M^−1^) requiring only low [porphyrin]/[DNA] ratios to reach saturation, followed by a weaker (K_b_ ~0.2 x 10^4^ M^−1^), secondary process requiring a much higher ligand concentration for saturation. Likewise, the binding of TMPyP4 to [d(T_4_G_4_)]_4_ in K^+^ solution was characterized by a single binding event with an association constant of 7.7 x 10^4^ M^−1^. In the presence of Na^+^ ions, the affinity of the [d(T_4_G_4_)]_4_ toward the TMPyP4 is greatly enhanced, indeed, the association constants are 162 x 10^4^ and 4.4 x 10^4^ M^−1^ for the first and second binding event, respectively. Their data clearly suggest that metal ions can have a role in determining the binding properties and highlight that care should be taken in the studies of molecules designed as quadruplex-specific ligands.

Erra *et al.* studied the binding properties of TMPyP4 with the [d(AG_3_T)]_4_ and [d(TG_4_T)]_4_ quadruplex structures, two tetramolecular parallel-stranded G-quadruplexes formed by truncated sequences of human and *Oxytricha* telomeric DNA, respectively [41]. The binding data obtained by ITC reveal similar affinities (about 2 x 10^5^ M^−1^) in both cases, however, the TMPyP4/quadruplex binding stoichiometries are 1:2 for [d(AG_3_T)]_4_ and 2:1 for [d(TG_4_T)]_4_, respectively (Table 1). They speculated that the 2:1 porphyrin/quadruplex stoichiometry could be consistent with an interaction between the ligand molecules and the two external G-tetrad planes of [d(TG_4_T)]_4_, whereas, the 1:2 stoichiometry suggests that one TMPyP4 molecule bridges two [d(AG_3_T)]_4_ quadruplexes. Their results suggest that, despite equal topologies of the quadruplex structures, the binding properties can be dependent on quadruplexes base composition.

Since the regulation of the structural equilibrium of G-quadruplex-forming sequences located in the promoter regions of oncogenes by the binding of small molecules has shown potential as a new opportunity for anticancer therapy, Freyer *et al.* investigated the interaction of TMPyP4 with the 27-mer quadruplex-forming sequence d[(TG_4_AG_3_)_2_TG_4_A_2_G_2_] from the promoter region of *c-myc* [42]. The study was complicated by the fact that the *c-myc* sequence is capable of forming multiple folded quadruplex structures (at least two conformers), so they can only report average thermodynamic parameters for binding of TMPyP4 to an unknown mixture of quadruplex species. They report a stoichiometry of 4:1 for the TMPyP4/*c-myc* quadruplex complex, and suggest the presence of two different binding modes, each consisting of two thermodynamically equivalent ligand-binding sites. The two highest-affinity sites exhibit a binding constant of 1.6 x 10^7^ M^−1^ and the two lowest-affinity sites exhibit a constant of 4.2 x 10^5^ M^−1^. Dissection of the free-energy change into the enthalpy- and entropy-change contributions for the two modes shows that for one mode the favorable free energy change is partly due to a favorable entropy change, while, for the other one the favorable free energy change is opposed by an unfavorable entropy contribution.

To evaluate the effect of loop orientation on quadruplex-TMPyP4 interaction, Arora and Maiti characterized the binding of cationic porphyrin to G-quadruplex structures present in the promoter regions of *c-myc* and *c-kit* oncogenes and in the human telomeric region [43]. The DNA quadruplexes used in the study were the one formed by the 21-mer human telomeric sequence d[G_3_(T_2_AG_3_)_3_]; the 22-mer d(G_4_AG_3_TG_4_AG_3_TG_4_) and the 21-mer d(G_3_AG_3_CGCTG_3_AG_2_AG_3_) from the promoter region of *c-myc* and *c-kit* (between −87 and −109 bp upstream of the transcription initiation site), respectively. The three G-quadruplexes differ in loop orientations. Additionally, the *c-myc* and *c-kit* G-quadruplex structures adopt an intramolecular parallel-stranded conformation, while the telomeric quadruplex adopts antiparallel conformation. The association of TMPyP4 with all the quadruplex structures exhibited negative changes in the binding enthalpies (Table 1). ITC experiments showed two independent binding processes, a stronger binding (10^7^ M^−1^) of TMPyP4, probably involving end stacking, and a weaker external binding (10^6^ M^−1^). Moreover, they revealed that the TMPyP4 molecule shows preferential binding to parallel G-quadruplex over antiparallel. Indeed, the binding affinity for parallel quadruplexes (10^7^ M^−1^) was one order of magnitude higher than for the antiparallel structure (10^6^ M^−1^). They concluded highlighting that differences in the loop orientation give rise to different conformations of quadruplex, which in turn govern the binding to small molecules, playing a central role in molecular recognition.

Distamycin A is a small molecule which binds with high affinity to duplex DNA [44], however, this molecule and some its analogues have also been shown to interact with DNA quadruplex structures [45–47]. Additionally, derivatives of distamycin have been reported to be inhibitors of the human telomerase enzyme [48]. These findings have stimulated calorimetric investigations aimed at characterizing interactions of distamycin and its derivatives with G-quadruplex structures. Particularly, our group have conducted ITC experiments to examine the binding of distamycin and its two carbamoyl derivatives (compounds **1** and **2**, containing four and five pyrrole units, respectively) (Figure 2) to the target [d(TG_4_T)]_4_ and d[AG_3_(T_2_AG_3_)_3_] quadruplexes from the *Tetrahymena* and human telomeres, respectively [49,50]. To evaluate any influence of the ions present in solution on the binding behavior, the interactions were examined using two different buffered solutions containing either K^+^ or Na^+^ at a fixed ionic strength. Experiments revealed that distamycin and compound **1** bind the investigated quadruplexes in both solution conditions, whereas compound **2** appears to have a poor affinity in any case. The thermodynamic data determined by ITC revealed that all the interactions are entropically driven processes and that the presence of different cations in solution affects the stoichiometry and thermodynamics of the interactions (Table 1). Interestingly, ITC measurements showed that the binding of distamycin to [d(TG_4_T)]_4_ in K^+^ solution is characterized by two distinct binding events, each involving two drug molecules, to give a final 4:1 complex. Furthermore, the thermodynamic parameters (entropically driven process) suggested that the distamycin interacts with the grooves of the [d(TG_4_T)]_4_ quadruplex. All these findings were confirmed by the NMR structure of the complex which shows two distamycin dimers bond simultaneously to two opposite grooves of the quadruplex [51]. These results encourage the design and the study of new quadruplex groove binders, a not yet fully developed field.

Perry *et al.* were the first to perform an ITC study concerning the quadruplex-interactive agents [52]. They were encouraged from a previous study where they demonstrated telomerase inhibition by a 2,6-diamidoanthraquinone derivative and presented evidence that the mechanism of action involved binding to and resultant stabilization of G-quadruplex structures [53]. They used ITC to prove and then to quantify the interaction of two selected regioisomers (1,4- and 2,7-difunctionalised amidoanthracene-9,10-diones) (compounds **3** and **4**, Figure 2) with the human telomeric sequence d[AG_3_(T_2_AG_3_)_3_] under conditions shown to favor G-quadruplex formation. In parallel with the telomerase inhibition activities exhibited by the two compounds, both isomers were shown to bind to G-quadruplex structure with a stoichiometry of 1:1 and with similar affinity (in the range 5 ÷ 8 x 10^4^ M^−1^) (Table 1). They concluded that the positional placement of the substituent dictates the exact mode of binding to G-quadruplexes (and thus the thermodynamic parameters), but it does not necessarily affect their ability to inhibit telomerase.

**Figure 3.**
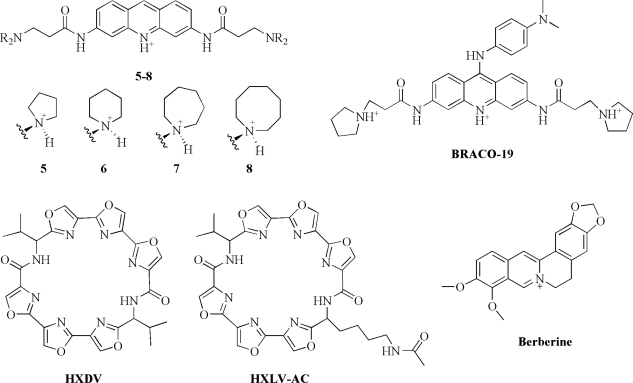
Chemical structures of quadruplex-interactive compounds studied by ITC.

To identify other suitable G-quadruplex-interactive compounds with improved activity against telomerase and superior physico-chemical properties (such as aqueous solubility), Read *et al.* performed a calorimetric study on small molecules with the acridine moiety, substituted in a manner analogous to the anthraquinones [54]. They investigated telomerase inhibitors based on the 3,6-disubstituted acridine chromophores (compounds **5**–**8**, Figure 3) to find structure-activity relationships between biological activity and substituent group size. The binding data obtained by ITC for the titration of human telomeric quadruplex d[AG_3_(T_2_AG_3_)_3_] with four members of the acridine series are collected in Table 1. The binding stoichiometry of the four complexes is 1:1 and the ranking order of binding enthalpy changes (in the range −6.9 ÷ −60.8 kJ mol^−1^) found in calorimetric studies is in agreement with the biological activity measured *in vitro.* They found that the compound with pyrrolidine end groups had the highest affinity to the human G-quadruplex structure. The authors suggested that these acridine-based compounds interact with the outer G-tetrads of the quadruplex structures through stacking interactions and that the cationic center incorporated into the aromatic moiety of the compounds confines the chromophore to a specific position, the center of the G-tetrad, where the oxygens of the four guanines generate an area of partial negative charge, strongly attracting partial positive charges and formally protonated species. Moreover, they suggested that the interactions of the protonated side chains with the negative phosphates are an important factor in the ability of these compounds to bind to DNA quadruplexes. In fact, as suggested by the ITC data, increasing the steric bulk around the nitrogen of side chains could shield it from participating in effective electrostatic interactions and also sterically restricts the formation of anchoring hydrogen bonds.

White *et al.* used ITC to study the binding of the trisubstituted acridine BRACO-19 (9-[4-(*N*,*N*-dimethylamino)phenylamino]-3,6-bis(3-pyrrolodinopropionamido) acridine) (Figure 3) to the human telomeric d[AG_3_(T_2_AG_3_)_3_] quadruplex [55]. BRACO-19 is among the most potent G-quadruplex-interactive compounds inhibitor of human telomerase described so far. It has nanomolar potency against telomerase and low nonspecific cytotoxicity. BRACO-19 has also shown to inhibit growth and to induce senescence in cancer cell lines. Moreover, it possesses a significant antitumor activity *in vivo* [56]. They found that the type and position of substituents on the acridine ring provide to the trisubstituted acridine BRACO-19 an exceptional quadruplex binding affinity. Indeed, they found that the magnitude of the binding constant is too large to be studied by ITC and they determined accurately only the enthalpy value (ΔH° = −34.7 kJ mol^−1^) (Table 1). Moreover, they observed two binding events in the ITC experiments at high concentrations of ligand. Based on the Gibbs energy (ΔG° = −42.3 kJ mol^−1^) calculated from equilibrium constants obtained by surface plasmon resonance (SPR) measurements and Δ_b_H° from ITC, they calculated a TΔ_b_S° value, for the strong binding site, of about 7.5 kJ mol^−1^. The BRACO-19 interaction with the human telomeric G-quadruplex is thus principally enthalpically driven with a smaller favourable contribution from the entropic term.

Another promising class of anticancer agents that target G-quadruplex DNA are the oxazole-containing macrocycles, which include the natural product telomestatin. Telomestatin (a heptaoxazole-containing macrocycle) has shown apoptotic activity in cancer cells and, very interestingly, it exhibits a high degree of selectivity for G-quadruplex relative to duplex DNA [57]. To develop new oxazole-containing ligands, Pilch *et al.* have characterized the binding of two synthetic hexaoxazole-containing macrocyclic compounds (HXDV and HXLV-AC, Figure 3) to the intramolecular G-quadruplex structural motif formed by human telomeric DNA in the presence of K^+^ ions [58]. Aside from six oxazole moieties, one of these compounds (HXDV) also contains two valine residues, while the other (HXLV-AC) contains one valine residue and one lysine residue in which the 4-aminobutyl side chain has been N-acetylated. Interestingly, both compounds exhibit cytotoxic activities versus human lymphoblast and oral carcinoma cells, with associated IC_50_ values ranging from 0.4 to 0.9 mM.

They used ITC also to evaluate the selectivity of the two compounds for G-quadruplex structures over duplex DNA. The ITC profiles for the titration of a duplex DNA into either buffer alone or buffer containing a ligand were essentially identical, indicating that the compounds bind solely to the quadruplex nucleic acid form, but not to the duplex. Binding to the quadruplex is associated with a stoichiometry of two ligand molecules per DNA molecule. The enthalpy changes for the binding are negative (favorable) in both cases, but, for both compounds, the binding is principally entropically driven (Table 1). ITC data revealed that HXDV and HXLV-AC bind to the quadruplex structure with association constants of 3.0 x 10^5^ and 5.5 x 10^5^ M^−1^, respectively. These results suggest that substitution of one valine functionality in HXDV with an *N*-acetylated lysinyl moiety affords a modest enhancement in affinity for human telomeric quadruplex DNA. A comparison of the enthalpy changes associated with the binding of HXLV-AC and HXDV indicates that the binding of HXLV-AC is slightly favored by enthalpic contribution, while the entropic contributions to the binding of both compounds are similar. These observations may reflect enhanced van der Waals and/or hydrogen bonding contacts afforded by the *N*-acetylated lysinyl moiety of HXVL-AC, since such contacts are typically manifested enthalpically.

Recent studies have shown that berberine (Figure 3) and some its analogues bind to telomeric G-quadruplex and inhibit the human telomerase activity [59]. Additionally, these molecules have exhibited high selectivity for G-quadruplex over duplex DNA. The aromatic moiety of berberine molecules plays a key role in quadruplex binding, making it an attractive scaffold to develop new ligands targeting G-quadruplex selectively. To obtain comprehensive knowledge of the interaction of this scaffold, Arora *et al.* performed an ITC study to obtain thermodynamic details of the interaction between berberine and human telomeric d[AG_3_(T_2_AG_3_)_3_] quadruplex (Table 1) [60]. Calorimetric titrations were performed at different temperatures and, at all the studied temperatures, the binding enthalpies were found to be negative, with their magnitude increasing with an increase in temperature. Through the temperature dependence of ΔH°, they obtained the heat capacity change (ΔC_p_) associated with berberine binding to quadruplex (−0.4 kJ mol^−1^ K^−1^), which falls within a range that is frequently observed for nucleic acid–ligand interactions. In all cases, the stoichiometry was found to be one mole of ligand per mole of quadruplex. The authors, on the basis of calorimetric results, ruled out the intercalative as well as minor groove binding mode and they suggested that berberine binds to quadruplex by stacking on the terminal G-tetrad of the quadruplex.

Recently, the group of Prof. Graves reported an ITC study of the interaction between actinomycin D and d[AG_3_(T_2_AG_3_)_3_] human telomeric sequence, to provide key insights into the thermodynamic features that characterize the binding reaction [61]. Actinomycin D is an anticancer agent that consists of a heterocyclic phenoxazone ring that serves as the interacalative portion of the ligand and two cyclic pentapeptide side chains that have extensive interactions within the minor groove of duplex DNA. It is well-known that actinomycin D is able to bind to duplex DNA containing G-tracts [62]; in that work, they have demonstrated the ability of actinomycin D to interact also with the G-quadruplex DNA structures. They performed the experiments in two different solutions containing either K^+^ or Na^+^ ions to investigate the binding of the drug to both the Na^+^ and K^+^ structural isoforms of the G-quadruplex DNA. The thermodynamic parameters for the binding of drug to both the Na^+^ and K^+^ forms of the G-quadruplex DNAs are quite similar, regardless of the initial starting structural form (Table 1). The binding of actinomycin D to the G-quadruplex DNAs is characterized by an association constants of approximately 2 x 10^5^ M^−1^. They observed that the formation of the drug-quadruplex complex for both the Na^+^ and K^+^ forms is enthalpically driven, with binding enthalpy changes of approximately −29 kJ mol^−1^. Interestingly, stoichiometries of approximately 0.5 (actinomycin D per quadruplex) were observed for both the Na^+^ and K^+^ quadruplex complexes with actinomycin D, indicating that one drug molecule could bridge two quadruplex DNA structures to form a 2:1 complex. These results support the hypothesis that the phenoxazone ring of actinomycin D stacks to a terminal G-tetrad of the quadruplex and that a second quadruplex structure stacks its terminal G-tetrad to the opposite face of the phenoxozone ring, resulting in the 2:1 binding stoichiometry.

General comments are required on both drug and quadruplexes involved in the studies reported above. The first comment is on the choice of the drugs, many of those were found to inhibit telomerase activity using the telomeric repeat amplification protocol (TRAP assay). That methodology was harshly criticized by Mergny and coworkers, they showed that TRAP is inappropriate for the determination of telomerase inhibition by quadruplex ligands, even combined with PCR controls, due to overestimated inhibitory effects [63]. However, all the drugs selected by TRAP assay have been shown to strongly interact with the quadruplex structures by ITC. This suggests that the combination of the TRAP and the ITC results could be useful to identify potential anticancer agents. The second comment is on G-quadruplex topologies depending on sequence, length and cations effects. The presence of multiple conformations in solution could invalidate the thermodynamic parameters extracted by ITC measurements. An example is represented by the human telomeric sequences. Indeed, several papers on this topic, published earlier than 2006 do not consider the polymorphism of those sequences, which was highlighted later, causing a poor interpretation of the phenomenon. In many cases, as the previous one, the assumption that the drug bind to a main target conformation could help to bypass the problem.

The final aim is to obtain a complete picture of ligand binding mode, based on the thermodynamic parameters, in analogy to the Chaires work on duplex DNA-ligands interactions [64]. Unfortunately, the ITC data are collected in different solution conditions and the affinity data are often hard to compare, therefore that aim is not fully achieved to date.

## 3. Protein Interactions

Aptamers are nucleic acid-based molecules that specifically bind to molecular targets (proteins, nucleic acids or small molecules). They are selected *in vitro* by SELEX (Systematic Evolution of Ligands by Exponential Enrichment), a combinatorial chemistry methodology based on oligonucleotide libraries which are screened for high-affinity binding to a given target [65]. High-affinity ligands can be isolated from the library using iterative rounds affinity-based enrichment, alternating with oligonucleotide amplification. Aptamers based on G-quadruplex motif have been proved to be useful tools aimed at binding and inhibiting particular proteins [66].

The best known aptamer capable of forming a G-quadruplex structure is TBA (Thrombin Binding Aptamer) [67]. TBA is based on a single-stranded 15-mer DNA of sequence d(G_2_T_2_G_2_TGTG_2_T_2_G_2_) which forms an unimolecular quadruplex in solution, arranged in a chair-like structure, consisting of two G-tetrads connected by two TT loops and a single TGT loop (Figure 1f) [68]. The TBA binds to thrombin with high-affinity and inhibits the thrombin-catalyzed fibrin clot formation [69]. Quadruplex-forming oligonucleotides have also resulted to be potent inhibitors of the HIV-1 integrase, the enzyme responsible for the insertion of viral DNA into the host genome [70]. Furthermore, an aptamer selected as inhibitor of human RNase H1 activity was found to fold into a unimolecular G-quadruplex consisting of a stack of two G-tetrads flanked by a stem formed by base pairing of the 5′ and 3′ tails of the oligonucleotide [71]. In addition, the existence of several G-rich regions with the potential to form G-quadruplex structures within human genome, implies that G-quadruplexes may play a role in a number of biological events, and proteins could also participate in these events. For example, some proteins bind selectively and tightly to G-quadruplexes, others promote the formation of G-quadruplex structures or act to unwind them [72].

Despite the fact that several proteins have been shown to interact with G-quadruplex structures, there are rather few studies aimed at understanding the energetic bases of such interactions. Recently, some of us have focused on the study of the binding of TBA and a modified TBA (mTBA) to the thrombin, to assess the binding stoichiometry and to gain information on the thermodynamic of interaction [73]. The mTBA has the sequence d(^3’^GGT^5’^-^5’^TGGTGTGGTTGG^3’^) containing a 5’-5’ site of polarity inversion [74]. It was designed and synthesized with the aim of improving the biological and biophysical properties of the natural thrombin aptamer. ITC measurements demonstrated that the binding of TBA and mTBA to thrombin is exothermic in nature and that both aptamers bind with a stoichiometry of 1:2 (aptamer/protein). The values of the binding constants and the Gibbs energy changes indicate that the associations are strongly favored, at 25°C, from a thermodynamic point of view and that the investigated aptamers bind to thrombin with different affinity. Indeed, the equilibrium constant for the interaction of mTBA with thrombin (K_b_ = 4 x 10^7^ M^−1^) is about one order of magnitude greater than that for the TBA-thrombin interaction (K_b_ = 3 x 10^6^ M^−1^). The values of Δ_b_H° and Δ_b_S° show that, in both cases, the binding processes are enthalpically driven; however, the interaction of mTBA with thrombin is associated with a larger favorable enthalpy (ΔH° = −160 kJ mol^−1^) as compared to TBA (ΔH° = −110 kJ mol^−1^). ITC data did not allowed us a distinction between the two sites because the titration data do not have distinct energetic profiles; however, the thermodynamic data reveal that mTBA has, on average, a higher affinity for thrombin and that its interaction is associated with a larger favorable enthalpy change.

Chen *et al.* reported a thermodynamic characterization of specific interactions between the human Lon protease (hLon) and G-quartet DNA [75]. Lon is an ATP-dependent protease that has multiple cellular functions, one of them is the binding to DNA [76–78]. Previous studies have suggested that hLon binds preferentially to a G-rich single-stranded DNA sequence overlapping the light strand promoter of mitochondrial DNA [79]. This 24-mer sequence d(A_2_TA_2_TGTGT_2_AGT_2_G_6_TGA), referred to as LSPas, contains six contiguous guanine bases. On the bases of electrophoretic and CD data, the authors suggested that LSPas forms intermolecular parallel G-quartet structures in Na^+^ solutions, similarly to the 8-mer d(TG_6_T). They used ITC to investigate the energetics of hLon interaction with LSPas and d(TG_6_T) over a temperature range from 10 °C to 30 °C. The binding of hLon to LSPas and d(TG_6_T) showed similar association constants (~105 M^−1^) and free energy changes. The binding strength seems to be weak, but reasonable because hLon most likely functions as a regulatory protein. Interestingly, the experiments revealed that hLon binding to LSPas is primarily driven by enthalpy change associated with a significant reduction in heat capacity (−2.5 kJ mol^−1^ K^−1^). The free energy change corresponding to the K_b_ was not susceptible to temperature changes, as a result of strong enthalpy–entropy compensation. The thermodynamic data revealed that, as for the binding to LSPas, hLon binding to d(TG_6_T) was exothermic, and exhibited moderate temperature dependence. However, in contrast to what was observed with LSPas, the heat capacity change for hLon binding to d(TG_6_T) was positive and relatively small (about 0.3 kJ mol^−1^ K^−1^). They deduced that the small change in heat capacity for the binding of hLon to d(TG_6_T) mainly accounts for the extent of dehydration, whereas the large negative heat capacity change associated with the binding to LSPas reflects a considerable involvement of hydrophobic interactions, suggesting the possibility that the association might be coupled with local folding of a number of amino acid residues.

The number of papers on the quadruplex interacting proteins is still limited probably due to the restricted examples found so far. However, the results reported here clearly demonstrate that the topic is rather intriguing and promising with a very high emerging interest, essentially connected to the aptamer design and their application. In this field, ITC represents a powerful tool to illuminate on the recognition processes governing the protein-quadruplex interactions. Particularly, the analysis of the thermodynamic contributions to the interaction could be useful to carry out chemical optimizations to enhance the affinity for a target protein.

## 4. Ion Interactions

As highlighted in the introduction, the coordination of cations is of fundamental importance in quadruplex formation. Indeed, these structures usually do not form in the absence of such ions. Generally, the cations are coordinated to O6 carboxyl oxygen of guanine bases between the planes of adjacent G-tetrads. Experiments have demonstrated that the G-quadruplexes are stabilized by the alkali series in the following order: K^+^>>Na^+^>Rb^+^>NH_4_+>Cs+>>Li+, and for the alkaline earth series in the order: Sr^2+^>>Ba^2+^>Ca^2+^>Mg^2+^ indicating that the atomic radii of 1.3Å of potassium and strontium fit best in the coordination site between adjacent G-tetrads [80,81].

ITC is most commonly used for binding studies, but the technique has been recently used to study the enthalpy of G-quadruplex folding. In this application, unstructured oligonucleotides were mixed with excess cation solutions in the calorimeter to monitor the total enthalpy of folding.

Kankia and Marky have investigated the folding of TBA into the G-quadruplex structure induced by the interaction of monovalent and divalent cations [82]. They found that in the presence of Rb^+^, NH_4_+, Sr2+, or Ba2+*,* the oligonucleotide folds into stable intramolecular G-quadruplex, similar to the one observed in the presence of K^+^. In particular, they used ITC to measure the heats of complex formation at 10 °C and 20 °C. They injected aliquots of cations into a Cs^+^-TBA (unfolded quadruplex) solution, and measured the heat changes accompanying quadruplex formation and TBA-cation binding. They found that the binding of a cation to the aptamer in the Cs^+^ form is, in general, accompanied by exothermic heats. The enthalpies at 20 °C range from −102.5 kJ mol^−1^ (K^+^) to −69.9 kJ mol^−1^ (Sr2^+^), while much lower exothermic heats are obtained at 10 °C. They suggested that the large difference between the enthalpies of K^+^ and Sr^2+^ complexes can be ascribed to different hydration contributions, that may arise from differences in both their hydration states and the actual release of electrostricted water upon binding to the aptamer to form the complex.

In another article, Kankia *et al.* studied the effect of human immunodeficiency virus type 1 nucleocapsid protein (NC) on the thermodynamic properties of the TBA quadruplex [83]. NC is a nucleic acid chaperone that catalyzes the rearrangement of nucleic acids into their thermodynamically most stable structures. NC was shown to preferentially recognize the intermolecular G-quadruplex structures [84]. The authors measured by ITC the effect of NC on the stabilization/destabilization of the quadruplex formed in the presence of different cations. Similarly to the experiments described above, the oligonucleotide solution (Cs^+^-TBA) in the absence or in the presence of NC protein was placed in the reaction cell, and titrated with K^+^ or Sr^2+^. A binding stoichiometry of 3:1 K^+^ cations per quadruplex was determined for solutions of TBA both alone and in the presence of NC. Interestingly, a strong decrease in quadruplex formation enthalpy was observed in the presence of NC. The ΔH value is reduced to ~70% of the one measured in the absence of NC. Moreover, the addition of NC results in a 59.4 kJ mol^−1^ increase in TΔS, which suggests that NC entropically stabilizes quadruplex formation. Thus, NC induced enthalpic destabilization (63.6 kJ mol^−1^) is almost compensated by entropic stabilization, resulting in only 4.2 kJ mol^−1^ net reduction in overall free energy of folding. Binding of Sr^2+^ ions to TBA reveals a stoichiometry of 1:1. As in the case of K^+^-TBA, the presence of NC does not affect binding stoichiometry, but the enthalpy of Sr^2+^-TBA formation is strongly affected by the presence of NC. Indeed, NC enthalpically destabilizes (by 37.6 kJ mol^−1^) and entropically stabilizes (by 33 kJ mol^−1^) the Sr^2+^-TBA quadruplex, resulting in a destabilization free energy of 4.6 kJ mol^−1^, which is similar to the value determined for K^+^-TBA. Hence, the thermodynamic data have revealed an enthalpically driven destabilization of both quadruplexes upon NC interaction, probably due to unstacking of the G-quartets upon protein binding.

Recently, Majhi *et al.* employed ITC to explore the temperature dependence of the enthalpy of formation for three unimolecular G-quadruplexes in the presence of excess concentrations of either Na^+^ or K^+^ [85]. They investigated three biologically relevant DNA G-quadruplex structures: the TBA, the aptamer PS2.M of sequence d(GTG_3_TAG_3_CG_3_T_2_G_2_) (which shows peroxidase activity when complexed with hemin) [86], and the human telomeric sequence d[AG_3_(T_2_AG_3_)_3_]. They performed ITC experiments adding small volumes of DNA solution to a solution containing salt in excess and measured the enthalpy of quadruplex formation at different temperatures. The results show that quadruplex folding is accompanied by a temperature dependent change in enthalpy. In particular, in all three cases the ΔH_unfold_ (= −ΔH_fold_) is endothermic over the temperature ranged studied, but in each case, the magnitude of the heat change increases as the temperature increases, indicative of ΔC_P_ > 0. Interestingly, both TBA and PS2.M show a linear dependence of ΔH_unfold_ as a function of temperature, while those for human telomeric quadruplex reveal a nonlinear behavior. From the linear temperature dependence of the enthalpy of unfolding, they estimated the change in heat capacity related to unfolding of TBA (2.8 kJ K^−1^ mol^−1^) and PS2.M (3.5 kJ K^−1^ mol^−1^); from the nonlinear temperature dependence accompanying unfolding of human telomeric quadruplex they estimated a range of heat capacities (4.3 ÷ 10.8 kJ K^−1^ mol^−1^). The ΔC_P_ values reported (about 1.4 kJ K^−1^ mol^−1^ per tetrad) are considerably larger than those estimated by DSC measurements (about 0.4 kJ K^−1^ mol^−1^ per tetrad), suggesting a significant role for contributions from solvent exposure effects coupled to processes involving stacking in the unstructured strands.

Usually, a biological system behaves in different ways according to the bulk conditions and often it is important to describe those differences. ITC could be considered as one of the best choice to investigate many conditions: buffers, the presence of molecular crowding agents, the presence of different cations. Among the cations above investigated, potassium and sodium ions are certainly the most relevant for their abundance in the biological fluids. As previously shown K^+^ and Na^+^ induce different conformations, in solution, on the telomeric or non-telomeric sequences, and quite often the resulting structures show different energetics of the folding processes.

Since the potassium ions are the most abundant in the cells, the ITC study should be carried out in the presence of K^+^ cations, or more interestingly, ITC study could be performed in a mixture of potassium and sodium cations in according to the physiological conditions.

## 5. Complementary Strand Interactions

The structural competitive mechanism between the G-quadruplex and the Watson-Crick duplex is extremely significant due to its biological implications. Several ITC studies of the interaction between G-quadruplex structures and complementary strands have been performed to clarify the factors dominating the competitive equilibrium between these multi-stranded structures. Interestingly, the cytosine-rich strands complementary to guanine-rich quadruplex-forming sequences can in turn form a self-associated structure called I-motif [87]. The I-motif is a tetrameric structure formed by two intercalated parallel duplexes containing hemiprotonated C.C+ base pairs, that could have, as G-quadruplex structures, a potential biological relevance.

Li *et al.* investigated the effects of pH and cation species in solution on duplex formation by the G-quadruplex d[G_3_(T_2_AG_3_)_3_] and its complement, the I-motif of sequence d[(C_3_TA_2_) _3_C_3_] [88]. The ITC experiments confirmed the duplex formation mixing the G-rich strand with its complementary strand. The binding constants showed that the competitive equilibria depend significantly on the cation species and pH. Indeed, at pH 7.0, the binding stoichiometry between the two strands was approximately 1:1 in the presence of magnesium ion as well as in the presence of sodium ion, supporting the preference for duplex formation in the mixture of the G-quadruplex and I-motif at pH 7.0. The binding constant in the presence of magnesium (10 mM Mg^2+^) at pH 7.0 was 4 times larger than that in the presence of sodium (100 mM Na^+^) at same pH and about 100 times larger than that in the presence of Na^+^ but at pH 5.5, indicating that the acidic pH lessens duplex formation. Moreover, the duplex formation in the magnesium buffer is associated with a 1.4 times more favorable enthalpy that in the sodium buffer at pH 7.0. Both results suggest that magnesium ion stabilizes the duplex and favor its formation.

Similar studies were performed by Miyoshi *et al.* for the truncated sequence of *Oxytricha* telomeric DNA d(G_4_T_4_G_4_) and its complementary strand d(C_4_A_4_C_4_) [89]. To mimic the cellular environmental conditions and to investigate their effect on the structure and stability of the telomeric DNAs, they performed the experiments in a molecular crowding condition. Interestingly, they found that the structural polymorphism of the telomeric DNA is induced by molecular crowding and, in addition, they demonstrated by ITC that such condition prevents the duplex formation. On the other hand, the ITC measurements indicated the duplex formation between the G-rich and C-rich strands in the noncrowding condition. However, the binding stoichiometry between the two strands resulted to be 0.41, indicating other side reactions (triplex and/or quadruplex formation) along with the duplex formation during the titration experiments in the noncrowding condition.

To achieve a deeper understanding of the physico-chemical properties of unusual DNA structures and their interaction with complementary oligonucleotides, Lee *et al.* investigated the reaction of an I-motif, G-quadruplex, and triplex with their complementary strands by using calorimetric techniques [90]. Along with other unusual DNA structures, they used TBA to target two different complementary strands (d(C_2_ACAC_2_A_2_C_2_) and d(C_2_A_2_C_2_ACAC_2_A_2_C_2_)) to form a duplex with a dangling end (in the first case) and a fully complementary duplex (in the second case), and to determine the thermodynamic contributions favoring the formation of the products. Both TBA-complementary strands reactions yielded favorable, enthalpy driven, free energy contributions, indicating that both single strands are able to invade and disrupt the quadruplex structure, forming a stable product. The favorable heat for the reactions is due to the net compensation of an exothermic heat from the formation of base-pair stacks and the endothermic heats of breaking both a G-tetrad stack and base-base stacking of the loops of TBA. The enthalpies measured directly in ITC titrations for the formation of the duplex products are −242.7 and −351.5 kJ mol^−1^ for the interaction of TBA with d(C_2_ACAC_2_A_2_C_2_) and d(C_2_A_2_C_2_ACAC_2_A_2_C_2_), respectively. The increasing magnitude of the enthalpy value clearly indicates the higher number of base-pair stacks that are formed in the latter case.

This example clearly shows that ITC is an helpful methodology, that gives information on the enthalpy contribution to the global energetics of the duplex on quadruplex propensity. This permits to gain information on the number and the type of interactions involved in the recognition processes allowing a full description of complicated processes as the duplex-quadruplex conversion.

## 6. Conclusions

ITC is one of the most important technique to study quadruplex-ligand binding affinity and the only procedure able to resolve the enthalpic and entropic contributions of the binding process. The combination of the favourable/unfavourable values, of the enthalpic and the entropic terms is indicative of the binding mode of different ligands to quadruplex structures. This thermodynamic information, the so-called “thermodynamic signature”, synergistically with structural data, is useful in drug designing for quadruplex molecular scaffold. Since quadruplex-drug recognition is a hot research field and considering that the ITC is a straightforward method, researchers should be encouraged to produce an increasing number of ITC data to enlarge the picture of the thermodynamic signature, particularly when structural data are not available.

## Figures and Tables

**Table 1.** Thermodynamic parameters for ligands binding to G-quadruplex structures obtained by ITC at 25 °C.

**Ligand**	**G-quadruplex**	**Buffer**	**n**	**K_b_ (M^−1^)**	Δ**H° (kJ/mol)**	**ΔG° (kJ/mol)**	**TΔS° (kJ/mol)**	**Ref.**
TMPyP4	d(G_2_T_2_G_2_TGTG_2_T_2_G_2_)	K^+^	1	17.8 × 10^4^	−40.2	−30.1	−10.0	[40]
TMPyP4	d[AG_3_(T_2_AG_3_)_3_]	K^+^	1.9	2.8 × 10^4^	−17.6	−25.5	8.0	[40]
TMPyP4-1	d[AG_3_(T_2_AG_3_)_3_]	Na^+^	1.1	3.3 × 10^4^	−28.4	−25.9	−2.5	[40]
TMPyP4-2	d[AG_3_(T_2_AG_3_)_3_]	Na^+^	1.0	~0.2 × 10^4^	~−25	n.d.	n.d.	[40]
TMPyP4	[d(T_4_G_4_)]_4_	K^+^	2.9	7.7 × 10^4^	−38.1	−28.0	−10.0	[40]
TMPyP4-1	[d(T_4_G_4_)]_4_	Na^+^	1.1	162 × 10^4^	−28.0	−35.6	7.5	[40]
TMPyP4-2	[d(T_4_G_4_)]_4_	Na^+^	2.0	4.4 × 10^4^	−105.8	−26.3	−79.5	[40]
TMPyP4	[d(AG_3_T)]_4_	Na^+^	0.5	1.7 × 10^5^	−40.5	−30.0	6.9	[41]
TMPyP4	[d(TG_4_T)]_4_	Na^+^	2	2.5 × 10^5^	−23.9	−30.8	−10.6	[41]
TMPyP4-1	d[G_3_(T_2_AG_3_)_3_]	K^+^	1	4.0 × 10^6^	−7.9	−37.6	29.7	[43]
TMPyP4-2	d[G_3_(T_2_AG_3_)_3_]	K^+^	2	0.5 × 10^6^	−17.6	−32.6	15.0	[43]
TMPyP4-1	d(G_4_AG_3_TG_4_AG_3_TG_4_)	K^+^	1	7.0 × 10^7^	−31.4	−44.3	12.9	[43]
TMPyP4-2	d(G_4_AG_3_TG_4_AG_3_TG_4_)	K^+^	2	1.0 × 10^6^	−42.7	−34.3	−8.4	[43]
TMPyP4-1	d(G_3_AG_3_CGCTG_3_AG_2_AG_3_)	K^+^	1	1.5 × 10^7^	−12.6	−41.0	28.4	[43]
TMPyP4-2	d(G_3_AG_3_CGCTG_3_AG_2_AG_3_)	K^+^	2	1.0 × 10^6^	−33.1	−34.3	1.2	[43]
Distamycin-1	[d(TG_4_T)]_4_	K^+^	1.8	0.4 × 10^6^	−8.0	−32	24	[51]
Distamycin-2	[d(TG_4_T)]_4_	K^+^	4.2	4.0 × 10^6^	−10.0	−37	27	[51]
Distamycin	[d(TG_4_T)]_4_	Na^+^	1.0	0.2 × 10^6^	−14.0	−30	16	[49]
Distamycin	d[AG_3_(T_2_AG_3_)_3_]	K^+^	0.9	0.6 × 10^6^	−14.0	−33	19	[50]
Distamycin	d[AG_3_(T_2_AG_3_)_3_]	Na^+^	1.0	1.0 × 10^6^	−9.0	−34	25	[50]
**1**	[d(TG_4_T)]_4_	K^+^	1.9	2.0 × 10^6^	7.0	−36	43	[50]
**1**	[d(TG_4_T)]_4_	Na^+^	0.9	2.3 × 10^6^	10.0	−36	46	[49]
**1**	d[AG_3_(T_2_AG_3_)_3_]	K^+^	1.0	2.0 × 10^6^	9.0	−36	45	[50]
**1**	d[AG_3_(T_2_AG_3_)_3_]	Na^+^	0.9	6.0 × 10^6^	−18	−39	21	[50]
**3**	d[AG_3_(T_2_AG_3_)_3_]	K^+^	1.2	7.9 × 10^4^	−23.0	−28.0	5.0	[52]
**4**	d[AG_3_(T_2_AG_3_)_3_]	K^+^	1.3	5.1 × 10^4^	−42.3	−26.8	−15.5	[52]
**5**	d[AG_3_(T_2_AG_3_)_3_]	K^+^	1.1	9.7 × 10^4^	−60.8	−28.5	−32.3	[54]
**6**	d[AG_3_(T_2_AG_3_)_3_]	K^+^	1.1	1.4 × 10^4^	−20.9	−23.7	2.8	[54]
**7**	d[AG_3_(T_2_AG_3_)_3_]	K^+^	1.2	1.4 × 10^4^	−17.6	−23.7	6.1	[54]
**8**	d[AG_3_(T_2_AG_3_)_3_]	K^+^	1	0.5 × 10^4^	−6.9	−21.1	14.2	[54]
BRACO-19	d[AG_3_(T_2_AG_3_)_3_]	K^+^	2	n.d.	−34.7	−42.3*^a^*	7.5*^a^*	[55]
HXDV	d[T_2_G_3_(T_2_AG_3_)_3_A]	K^+^	2.2	3.0 × 10^5^	−7.1	−31.4	24.3	[58]
HXLV-AC	d[T_2_G_3_(T_2_AG_3_)_3_A]	K^+^	1.9	5.5 × 10^5^	−8.8	−32.6	23.8	[58]
Berberine	d[AG_3_(T_2_AG_3_)_3_]	K^+^	1	0.4 × 10^6^	−7.1	−32.2	25.1	[60]
Actinomycin D	d[AG_3_(T_2_AG_3_)_3_]	K^+^	0.6	2.3 × 10^5^	−28.0	−30.6	2.6	[61]
Actinomycin D	d[AG_3_(T_2_AG_3_)_3_]	Na^+^	0.5	2.1 × 10^5^	−30.5	−30.2	−0.3	[61]

^a^ These values were obtained from SPR equilibrium constant. n.d. = not determined.
